# Longer-term recurrence rate after low versus high dose radioiodine ablation for differentiated thyroid Cancer in low and intermediate risk patients: a meta-analysis

**DOI:** 10.1186/s12885-020-07029-3

**Published:** 2020-06-15

**Authors:** I. Vardarli, F. Weidemann, M. Aboukoura, K. Herrmann, I. Binse, R. Görges

**Affiliations:** 1 Department of Medicine I, Klinikum Vest GmbH, Knappschaftskrankenhaus Recklinghausen, Academic Teaching Hospital, Ruhr-University Bochum, Dorstener Str. 151, 45657 Recklinghausen, Germany; 2 Department of Nuclear Medicine, University Hospital Essen, Hufelandstraße 55, 45147 Essen, Germany

**Keywords:** Longer-term, Outcome, Radioactive iodine ablation, Differentiated thyroid carcinoma, Meta-analysis

## Abstract

**Background:**

Regarding the longer-term recurrence rate the optimal activity for the remnant thyroid ablation in patients with differentiated thyroid cancer (DTC) is discussed controversially. For the short-term ablation success rate up to 12 months there are already several meta-analyses. In this study we performed the first meta-analysis regarding the longer-term recurrence rate after radioactive 131-I administration.

**Methods:**

We conducted an electronic search using PubMed/MEDLINE, EMBASE and the Cochrane Library. All randomized controlled trials (RCTs) assessed the recurrence rate after radioactive iodine ablation in patients with DTC, with a follow-up of at least two years were selected. Statistics were performed by using Review Manager version 5.3 and Stata software.

**Results:**

Four RCTs were included in the study, involving 1501 patients. There was no indication for heterogeneity (*I*^*2*^ = 0%) and publication bias. The recurrence rate among patients who had a low dose 131-iodine ablation was not higher than for a high dose activity (odds ratio (OR) 0.93 [95% confidence interval (CI) 0.53–1.63]; *P* = 0.79). The mean follow-up time was between 4.25 and 10 years. The subgroup analysis regarding the TSH stimulated thyroglobulin values (< 10 ng/mL versus < 2 ng/mL versus ≤1 ng/mL) showed no influence on recurrence rate.

**Conclusions:**

For the first time we showed that the longer-term, at least 2-year follow-up, recurrence rate among patients who had 131-iodine ablation with 1.1 GBq was not higher than with 3.7 GBq.

## Background

Most cases of thyroid cancer are differentiated, with a high five-year survival rate of 90–95% [1, 2]. These patients commonly have total or near-total thyroidectomy followed by radioactive iodine (RAI) ablation and thyroid-stimulation hormone suppression therapy with levothyroxine [3]. In spite of lack of meta-analyses with longer-term follow-up data from RCTs in relation to recurrence rates, US and UK guidelines [4, 5] recommend a low radioactive iodine activity (1.1 GBq) in selected low-risk patients with DTC [6].

For the *ablation success rate up to 12 months follow-up* (comparing 1.1 GBq vs 3.7 GBq) in patients with DTC there are various meta-analyses with controversial results [7–13]. Three of these meta-analyses recommend low dose activity [7, 8, 10]: Cheng et al. analyzed 6 RCTs involving 1809 patients. There was no statistically difference in successful ablation (1.1 GBq vs 3.7 GBq radioiodine) (OR 0.91 [95% CI 0.79–1.04]; *P* = 0.15), and they found with 1.1 GBq significant benefits in reducing adverse effects [7]. Ma et al. included three RCTs (637 patients with DTC). On the basis of diagnostic scans they found no statistically significant differences between 1.1 GBq and 3.7 GBq radioiodine ablation (OR 0.85 [95% CI 0.49–1.47]; *P* = 0.56) with significant reduction in adverse events [8]. Valachis et al. analyzed eight randomized trials with 1772 patients. They reported no statistically difference between 1.1 GBq and 3.7 GBq (risk ratio (RR) 0.94 [95% CI 0.85–1.04]; *P* = 0.21) [10]. Two of the meta-analyses recommend high dose activity [11, 12]: Song et al. included seventeen RCTs, involving 3737 patients. They showed that ablation with 3.7 GBq had statistically significant higher (11%) successful ablation rate than 1.1 GBq (RR 0.89 [95% CI 0.81–0.97]; *P* = 0.008) [11]. Shengguang et al. analyzed nine RCTs (with 1769 patients). They found that the ablation success was 5% lower using 1.1 GBq compared with 3.7 GBq (OR 0.95 [95% CI 0.91–0.99]) [12]. Two remaining meta-analyses showed that it cannot be determined whether 1.1 GBq or 3.7 GBq should be used [9, 13]: Hackshaw included three RCTs with 148 patients. The ablation success was not significantly different when using 1.1 GBq compared with 3.7 GBq. They recommend large randomized trials to guide this issue [13]. Fang et al. included seven RCTs; they found no significant differences between 3.7 GBq and 1.1 GBq (RR 0.83 [95% CI 0.68–1.01]) [9].

Regarding the *longer-term recurrence rate* the optimal activity for the remnant thyroid ablation in patients with differentiated thyroid cancer (DTC) is discussed controversially.

To the best of our knowledge, in this study we performed the first meta-analysis regarding the longer-term recurrence rate after radioactive 131-I administration.

## Methods

The meta-analysis was performed according to the PRISMA guidelines [14]. The PRISMA check list is provided as Supplemental material [see Additional file 1].

### Data search and study selection

The electronic databases of PubMed/MEDLINE, EMBASE and Cochrane Library were systematically searched with the following *search strategies* (updated on January 11, 2020): PubMed/MEDLINE (http://www.ncbi.nlm.nih.gov/entrez/query.fcgi?DB=pubmed): ((“Thyroid Neoplasms/radiotherapy”[Majr] AND ablat*) OR (thyroid AND (cancer OR carcinoma))) AND (radioiodine OR radiotherapy) AND ablat* AND (long-term OR recurrence); EMBASE (http://ovidsp.dc2.ovid.com/sp-4.02.0b/ovidweb.cgi): (((Thyroid and neoplasm* and radiotherapy and ablat*) or (thyroid and (cancer or carcinoma))) and (radioiodine or radiotherapy) and ablat* and (long-term or recurrence)).mp. [mp = title, abstract, heading word, drug trade name, original title, device manufacturer, drug manufacturer, device trade name, keyword, floating subheading word, candidate term word]; The Cochrane Library (https://www.cochranelibrary.com/advanced-search): (Thyroid neoplasms or thyroid) and (cancer or carcinoma) and (radioiodine or radiotherapy) and ablat* and long-term and recurrence; without language and time restriction in any of these databases. Furthermore, references of retrieved studies were searched for eligible studies. Electronic archives of medical societies (Deutsche Gesellschaft für Nuklearmedizin e.V. (https://www.nuklearmedizin.de/jahrestagungen/abstr_online2019/abstract_search.php?navId=227) and Endocrine Society (https://www.endocrine.org/meetings/endo-annual-meetings) were also searched. Studies were included if they met the following inclusion criteria: randomized controlled trial (RTC); patients with differentiated thyroid carcinoma; comparison of low versus high radioiodine ablation activity; longer-term follow-up (at least 2 years after randomization); patients after thyroidectomy, near-total-thyroidectomy or subtotal thyroidectomy as initial ablation therapy; patients with ablative radioiodine therapy, post-operatively; initial assessment of the ablation success within three to 12 months post radioiodine ablation. Exclusion criteria were: Patients with local or regional metastases at inclusion in the study; patients with hemithyroidectomy; patients with medullary or anaplastic carcinomas; no data corning the endpoints; no comparison group available; no randomization performed; duplication of a study (in this case, inclusion of the study with the longest follow-up); only congress communication, not published as full-text paper; animal study.

### Data extraction and quality assessment

Two authors (IV and IB) independently reviewed all eligible articles and extracted the relevant data. In case of disagreement, after consultation with a third author (FW) regarding the eligibility, consensus was found. We used the Cochrane risk of bias tool to assess the risk of bias of each trial; following aspects were checked by two independent authors: random sequence generation (selection bias), allocation concealment (selection bias), blinding (performance bias), incomplete outcome data (attrition bias), selective reporting (reporting bias), and other source of bias. The two independent authors evaluated each item as unclear, high or low risk of bias [15].

### Statistical analysis

After the data extraction a meta-analysis was performed. Heterogeneity and publication bias of the included studies were checked; odds ratio for the primary endpoint was calculated.

The primary endpoint was defined as the recurrence rate between the low and high radioiodine activity, as defined by each eligible study. Recurrence was defined as pathologic findings, as defined by each study (e.g., fine needle aspiration (FNA), serum thyroglobulin (Tg), ultrasonography, diagnostic radioactive iodine scan, PET-Scan or MRI scan) during the follow-up; histologic findings were not defined as conditio sine qua non. Predefined secondary endpoints were: Successful ablation rate at first evaluation between low and high radioidine activity, as defined by each eligible study; definition of successful ablation at first evaluation: No uptake in WBS and/or Tg level < 2.0 ng/mL or < 10.0 ng/mL, as defined by each eligible study; early adverse effects (within 1 week after ablation) related to radioiodine ablation (including salivary dysfunction, neck pain, lacrimal dysfunction, nausea and any serious adverse events). Following subgroup analyses for the primary endpoint were predefined: method of TSH-stimulation (withdrawal versus rhTSH versus both); surgery method (total thyroidectomy vs near-total thyroidectomy vs subtotal thyroidectomy); preablation (131-I ablation) Tg measurements; preablation scan (e.g. Tc99m, 131-I < 2.0 mCi, 131-I ≥ 2.0 mCi); definition of successful ablation (e.g., Tg < 2.0 ng/mL & WBS, Tg < 10.0 ng/mL &WBS, WBS only); sample size, as applicable; patients with lymph node metastases included versus not included; country of origin (europe, asia, others); early adverse effects, if applicable.

In case of heterogeneity a meta-regression analysis (using Stata, Stata Corp, College Station, Texas) with following predefined covariates (potential confounders) was intended: method for TSH-stimulation (withdrawal vs rhTSH); surgery method (total thyroidectomy versus near-total thyroidectomy vs subtotal thyroidectomy); preablation (131-I ablation) Tg measurement; preablation scan; definition of successful ablation; sample size, as applicable; country of origin (europe, asia, others).

The meta-analysis was performed using Review Manager (RevMan) version 5.3. (Nordic Cochrane Center). For the calculation of effect size (odds ratio, 95% CI) we used the random effects model [16]. For the evaluation of heterogeneity Cochran‘s *Q* statistics [17] and the *I*^*2*^-statistic [18] were used; *P* > 0.1 and *I*^*2*^-statistic values less than 50% were considered as an indication of the lack of heterogeneity. For the assessment of publication bias we used funnel plot (using RevMan (Nordic Cochrane Center)).

## Results

### Study selection and characteristics

**Fig. 1:**
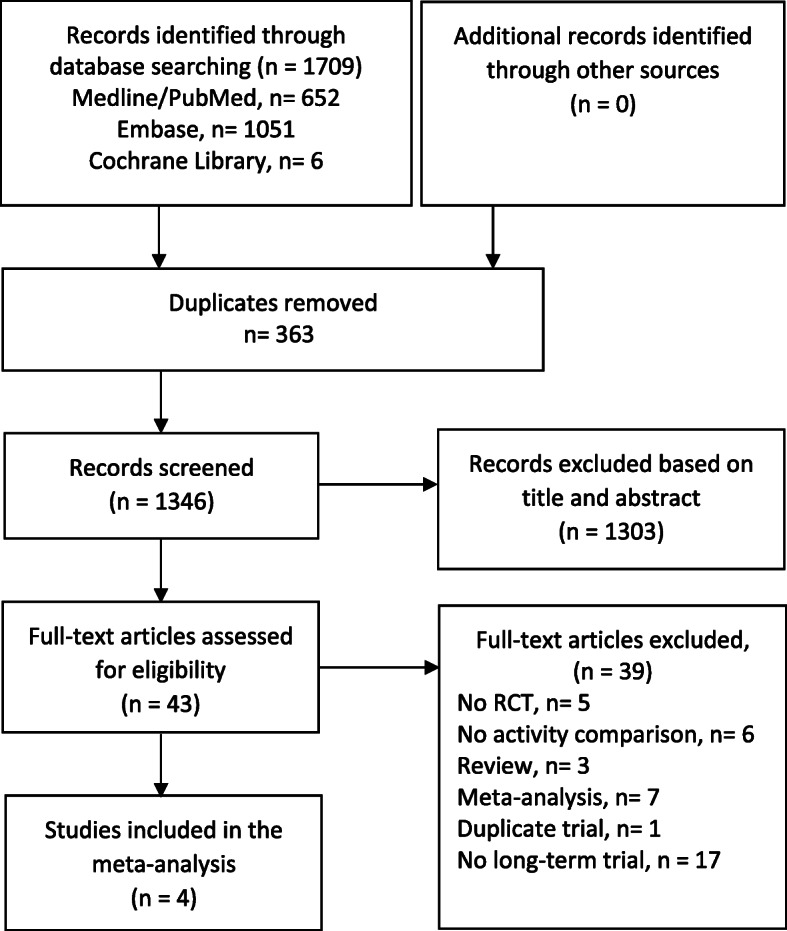
Flow chart showing the process for inclusion and exclusion of trials, according to the PRISMA guidelines

The literature search identified 1709 records with potentially relevant studies. As shown in Fig. 1 four RCTs met the inclusion and exclusion criteria which were included in the meta-analysis [3, 19–21]. The included studies had a total of 1501 patients. All of these studies were conducted prospectively. The detailed characteristics of the included studies are given in Table 1.

**Table 1:** Characteristics of the included studies

First Author Year	Country	Patients finally evaluated	Pathology DTC	TNM stage of included patients	Type of surgery	Activity [GBq] (number of patients)	Follow-up Time (years)	Definition of ablation success	Definition of recurrence
Mäenpää 2008 [19]	Finland (monocentric)	160	P / F	T?, any N, M0 (“patients with macroscopic inoperable locoregional disease were excluded”)	TT or NT	1.1 (*n* = 81) vs 3.7 (*n* = 79*) after WD (randomization: stratified according to the presence or absence of histologically verified cervical lymph node metastases)	Median 4.25 (51 months) (18–77 m)	4–8 months after RIT: 1) absence of abnormal uptake in a diagnostic whole body 131-I scan (185 MBq after WD), 2) Tg < 1 ng/mL) during both levothyroxine administration and TSH stimulation (WD or rhTSH), 3) absence of palpable metastases in the neck (neck US not mandated). All three conditions had to be met for ablation to be considered as successful.	SA; metastatic cervical lymph nodes were removed (=histology?); the 1.1 GBq group, n = 6; the 3.7 GBq group, *n* = 6. Three patients in the 3.7 GBq group and none in the 1.1 GBq group were diagnosed with distant metastases (Histology?) (*P* = 0.12). None died from thyroid cancer during follow-up.
Kukulska 2010 [20]	Poland (monocentric)	181 (86 + 95)	P / F	T ≥ 1b or Tx, any N, M0 (only patients with no evidence of persistent disease after TT and appropriate lymph node dissection)	TT (and in most cases lymph node dissect.)	1.1 (*n* = 86) vs 2.2 (*n* = 128) vs 3.7 (*n* = 95) (30 mCi vs 60 mCi vs 100 mCi) after WD	Median 10 (2–12)	12 months after RIT (after WD): 1) absence of thyroid bed uptake in 131-I neck scan, 2) stimulated Tg < 10 ng/mL. All two conditions had to be met for ablation to be considered as successful.	follow up; ultrasonography and radiological examinations and serum Tg level (on LT4-suppressive treatment), Histology not available
Schlumberger 2018 [21]	France (polycentric, 24 centers)	726	P / F (excluding aggressive histological subtypes)	T1 ≤ 1 cm with N1 or Nx, T1 > 1 cm with any N, T2 with N0, always M0	TT	1.1 (*n* = 363) vs 3.7 (*n* = 363) 359 after WD, 367 rhTSH (randomized)	Median 5.4 (0.5–9.2)	6–10 months after RIT (after rhTSH): 1) normal result on neck US and 2) Tg ≤ 1.0 ng/mL after stimulation (or a normal diagnostic iodine total-body san with 148-185 MBq (4-5 mCi) in patients with serum thyroglobulin antibodies)	follow up; Tg > 1 ng/mL on levothyroxine treatment was considered abnormal. Structural abnormalities on neck US were confirmed by FNA. No evidence of disease was defined as serum Tg ≤1 ng/mL on levothyroxine treatment and normal results on neck US when performed, but TSH stimulated serum thyroglobulin was not taken into account in this classification; Histology not available
Dehbi 2019 [3]	UK (polycentric, 29 centers)	434	DTC, no aggressive malignant variants	T1–3, any N, M0	TT or NT (with or without lymph node dissect.)	1.1 (*n* = 217) vs 3.7 (*n* = 217) 216 after WD, 218 rhTSH (randomized)	Median 6.5 y 78.4 months) (0.3–127 m)	6–9 months after RIT: serum Tg < 2.0 ng/mL and scan uptake < 0.1% (neck US not routinely used).	follow-up at annual clinical visits. Methods used to diagnose recurrence: serum Tg, neck US, diagnostic radioactive iodine scan, PET-scan, MRI scan; FNA (in some patients)

*DTC* Differentiated thyroid cancer. *P* Papillary carcinoma, *F* Follicular carcinoma. *TT* Total thyroidectomy, *NT* Near-total thyroidectomy. *RIT* Radioiodine therapy. *TSH* Thyroid stimulating hormone. *US* Ultrasonography. *SA* Structural abnormalities. *WD* ≥ 4 weeks levothyroxine withdrawal (or liothyronine for 14 days). *rhTSH* Recombinant human TSH. *FNA* Fine needle aspiration cytology. *Tg* Thyroglobulin. *2 patients received only 2220 MBq

### Risk of bias and publication bias

**Fig. 2:**
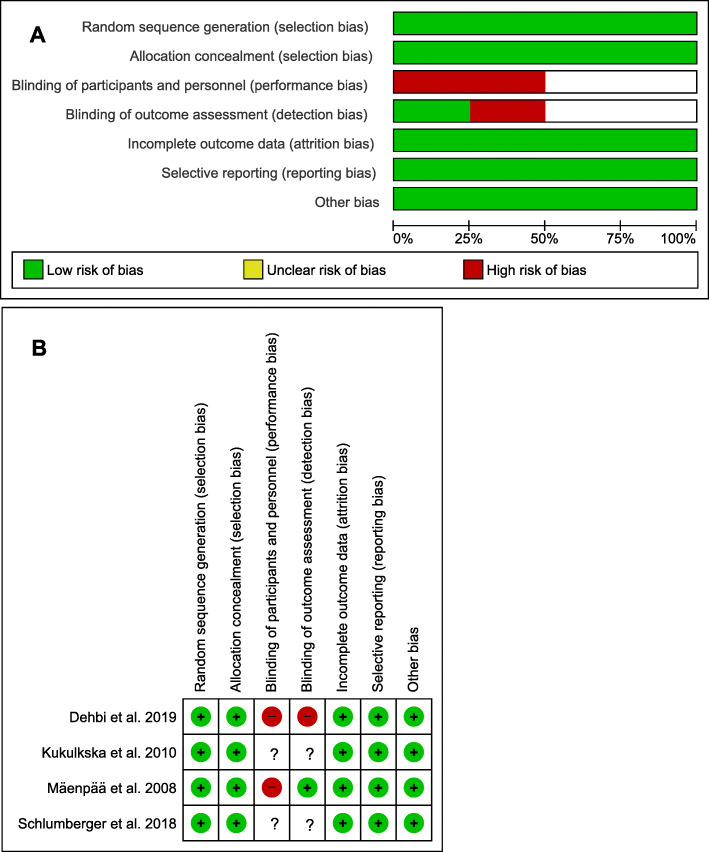
A: Risk of bias graph for all included studies. B: Risk of bias summary. “**+**” indicates a low risk of bias; “**-**“ indicates a high risk of bias; “**?**” indicates an unclear risk of bias

The risk of bias and quality of included studies are outlined in Fig. 2. Overall, the included studies were carried out well and had a relatively low risk of bias.

The funnel plot suggested no evidence for obvious publication bias [see Additional file 2]. Due to the small number of included studies, the Egger’s regression test was not performed.

### *Meta-*analysis

**Fig. 3:**
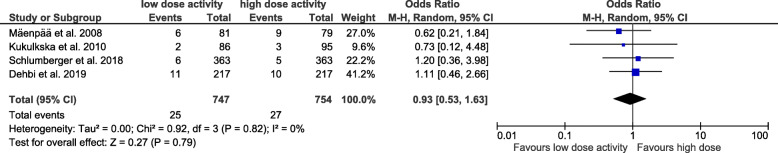
Comparison of longer-term disease recurrence rate between low-dose and high-dose 131-I activity, in all included studies

Even though there was no indication for heterogeneity (*I*^*2*^ = 0%) between the included studies, for the calculation of the effect size we used the random-effects model, as the test for heterogeneity often has a low power. Moreover, the effect sizes of the included trials can be seen having sampled from a distribution of effect sizes [22]. In our meta-analysis Tau^2^ is zero, reducing the random-effects analysis to the fixed effect analysis [22]. The included trials showed that the longer-term recurrence rate among patients who had low activity radioactive iodine ablation was not higher than for high dose (OR 0.93 [95% CI 0.53–1.63]; *P* = 0.79) (Fig. 3).

Mäenpää et al. showed in a randomized, open-label, single center study with 160 patients with papillary or follicular thyroid cancer after total thyroidectomy, comparing 1.1 GBq versus 3.7 GBq radioactive iodine activity, with a follow-up of 51 months (range18–77) that there is no conclusive evidence that 3.7 GBq activity is more effective for ablation of the thyroid remnant than 1.1 GBq activity. The 3.7 GBq activity was associated with more adverse effects [19].

Kukulska et al. showed in a randomized clinical trial with 309 patients with DTC (265 with papillary and 44 with follicular carcinoma) after total thyroidectomy and appropriate extent of neck lymph node dissection, comparing 30 mCi (1.1 GBq), 60 mCi (2.2 GBq) and 100 mCi (3.7 GBq) radioactive iodine activity, with a medial follow-up of 10 years [2–12] that no significant differences in the 5 year efficacy of thyroid remnant radioiodine ablation using 30, 60 and 100 mCi were observed [20].

Schlumberger et al. showed in a multicenter, randomized, open-label equivalence trial with 726 patients with low-risk differentiated thyroid cancer who had undergone total thyroidectomy, and a median follow-up since randomization of 5.4 years, comparing 1.1 GBq versus 3.7 GBq iodine-131-activity, that disease recurrence was not related to the strategy used for ablation, and stated that the data valid the use of 1.1 GBq radiodine-131 after rhTSH for postoperative ablation in patients with low-risk thyroid cancer [21].

Dehbi et al. showed in a non-inferiority, parallel, open-label, randomized controlled study with 438 patients with differentiated thyroid cancer after total or near-total thyroidectomy, comparing 1.1 versus 3.7 GBq radioactive iodine activity, that the recurrence rate among patients who had 1.1 GBq radioactive iodine ablation was not higher than that for 3.7 GBq; as providing further evidence in favor using low-dose radioactive iodine for treatment of patients with low-risk differentiated thyroid cancer. They found that the data indicate that recurrence risk was not affected by use of rhTSH [3].

**Fig. 4:**
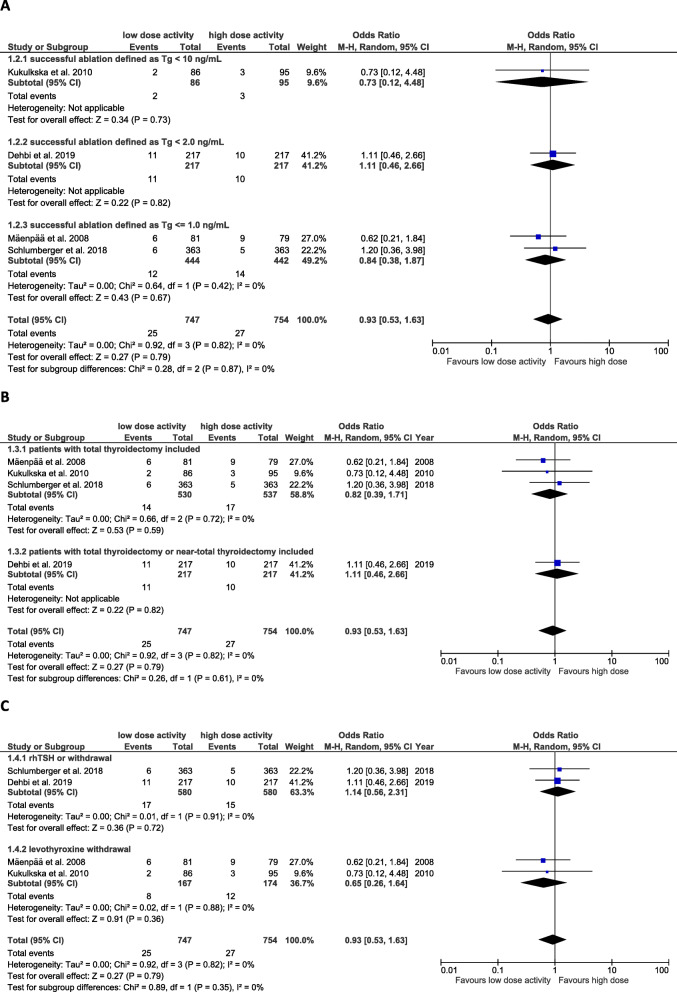
Comparison of longer-term disease recurrence rate between low-dose and high-dose 131-I activity. **a**: Subgroup analysis regarding ablation success definition. **b**: Subgroup analysis regarding type of surgery at inclusion. **c**: Subgroup analysis regarding stimulation method

The median follow-up time in the included studies in our meta-analysis was between 4.3 and 10 years (range 2–12). The ablation success was defined as Tg < 2 ng/mL or ≤ 1 ng/mL, respectively, in three trials [3, 19, 21] and as Tg < 10 ng/mL in one trial [20]. The subgroup analysis regarding the TSH stimulated thyroglobulin values (< 10 ng/mL versus < 2 ng/mL versus ≤1 ng/mL) showed no influence on longer-term recurrence rate (Fig. 4a).

The subgroup analyses regarding the type of surgery (total thyroidectomy versus total or near-total thyroidectomy) (Fig. 4b) and the stimulation method (rhTSH versus levothyroxine withdrawal) showed no influence on the longer-term recurrence rate (Fig. 4c).

All included trials were conducted with the possibility of patients with lymph node metastases in the included patients; two studies [19, 20] described the status as Nx, whereas in two other studies [3, 21] the possibility was clear described. All included studies were performed in Europe. Adverse effects were not adequately reported in each of the included studies. Thus, subgroup analyses regarding these parameters could not be performed.

## Discussion

Several meta-analyses with a short follow-up (no longer than 2 years) showed conflicting results. Some of these meta-analyses recommend the low-dose RAI ablation [7, 8, 10], other meta-analyses recommend higher ablation activities [11, 12], or failed to conclude which activity is the best in successful ablation rates [9, 13]. This is the first meta-analysis evaluating the longer-term recurrence rate after RAI ablation in patients with DTC. In our meta-analysis the follow-up time was between 2 and 12 years in range. Our results indicate that the longer-term recurrence rate among patients who had low radioiodine ablation activity (1.1 GBq 131-I) was not higher than those who had a higher activity (3.7 GBq 131-I).

The treatment decision for RAI ablation must be individualized based on the individual risk profile of the patient, balancing the risk and benefits [23]. A personalized postoperative approach for the management of DTC with low-risk status should be considered.

The feasibility of a randomized controlled trial investigating potential benefit of adjuvant radioiodine ablation in differentiated thyroid cancer has been frequently discussed; the sample size required to determine whether a mortality benefit exits with this intervention may not be feasible, especially given the rarity of thyroid cancer-related mortality in low-risk papillary cancer patients [23, 24]. However, a randomized controlled trial including a carefully stratified randomization strategy, with the outcome of recurrence may be feasible [25]. Large randomized controlled trials comparing any particular postoperative strategy, especially using recommended response criteria by American Thyroid Association (ATA), with the intention of modulating decision making on RAI remnant ablation or RAI treatment are needed [26].

According to the nine Martinique principles [27], the goals of 131-I therapy must be defined, as remnant ablation, adjuvant treatment, or treatment of known disease; the importance of evaluating postoperative disease status and multiple other factors beyond clinicopathologic staging assessments in 131-I, including serum Tg measurement, neck ultrasonography and diagnostic radioactive iodine scanning [4], therapy decision making should be described; it should be recognized that the optimal administered activity of 131-I adjuvant treatment cannot be definitely determined from the published literature. Until definitive data are available, selection of the administered 131-I activity for individual adjuvant treatment should be preferably based on multidisciplinary team management recommendations [27].

Various definitions of ablation success concerning stimulated Tg has been used in studies investigating the longer-term recurrence of DTC. According to the 2015 ATA management guidelines for differentiated thyroid cancer, after total thyroidectomy and radioiodine ablation, an excellent response was defined as TSH-stimulated Tg of < 1 μg/mL [4, 28]. Biochemical incomplete response was defined as TSH- stimulated Tg of ≥10 ng/mL; 20% of which develop structural disease, and less than 1% disease specific death [4]. In the primary studies, which we included in our meta-analysis, the ablation success was defined as TSH-stimulated Tg < 2 ng/mL or ≤ 1 ng/mL, respectively, in three trials [3, 19, 21] and as Tg < 10 ng/mL in one trial [20]; which is concurring with the 2015 ATA guidelines [4].

Kukulsa et al. [20] defined TSH-stimulated Tg of < 10 ng/ml (a gray zone) as successful ablation, in agreement with the ATA management guidelines, not as excellence response, but at least not as suspicious of biochemical incomplete response. Our subgroup analysis regarding the TSH-stimulated thyroglobulin values (< 10 ng/mL versus < 2 ng/mL versus ≤1 ng/mL) showed no influence on longer-term recurrence rate (Fig. 4). The 2015 ATA guideline recommend in low-risk and intermediate-risk patients (recommendation No. 63) who have had remnant ablation, measurement of Tg at 16–18 months after TSH stimulation to verify absence of disease (defined as excellence response) [4]. Hence, in further primary studies investigating the long-term recurrence of DTC, for TSH stimulated Tg a cut-off of < 1 ng/mL should be used.

In our study there are some limitations. First one is the small number of included trials. However, currently in the literature there exists only four trials, which meets our inclusion criteria. Second, we cannot exclude publication bias; the visual interpretation of funnel plots may be too subjective, and even in absence of asymmetry in funnel plot, bias cannot be excluded. We did not performed the Egger’s regression test, as it has a low power when the number of studies included is small [15]. Third, due to the small number of trials, the information given by the subgroup analyses is limited.

## Conclusions

Our results indicate that the longer-term (median follow-up period 4.3–10 years) recurrence rate among patients who had low radioactive iodine ablation was not higher than for high dose. However, because of the small number of published trials on this issue, further appropriate RCTs analyzing the long-term recurrence after DTC are necessary.

## Supplementary information

**Additional file 1 Table S1.** The PRISMA check list.

**Additional file 2 Figure S1.** Funnel plot of all included studies evaluating the longer-term recurrence in the low-activity versus high-activity 131-I group in patients with DTC, showing no indication for publication bias.

## Data Availability

Not applicable.

## Abbreviations

ATA: American thyroid association
CI: Confidence interval
DTC: Differentiated thyroid cancer
FNA: Fine needle aspiration cytology
GBq: Gigabecquerel
OR: Odds ratio
PRISMA: Preferred reporting items for systematic reviews and meta-analyses
RAI: Radioactive iodine
RCT: Randomized controlled trial
rhTSH: Recombinant human TSH
RIT: Radioiodine therapy
TSH: thyroid stimulating hormone
RR: Relative risk
Tg: Thyroglobulin.
US: Ultrasonography
