# Supplementary figures and images for: Longer-term recurrence rate after low versus high dose radioiodine ablation for differentiated thyroid Cancer in low and intermediate risk patients: a meta-analysis
Source: BMC Cancer. 2020 Jun 15;20:550. doi: 10.1186/s12885-020-07029-3 (PMC7296693; doi:10.1186/s12885-020-07029-3)

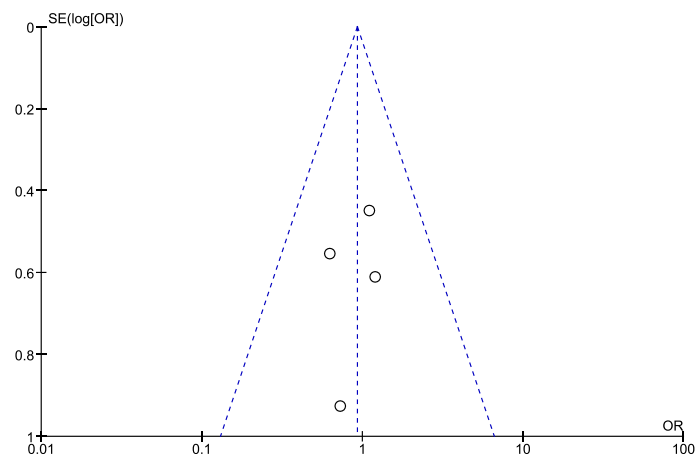

Supplement: Supplementary file 2 — Additional file 2 Figure S1. Funnel plot of all included studies evaluating the longer-term recurrence in the low-activity versus high-activity 131-I group in patients with DTC, showing no indication for publication bias. [file 12885_2020_7029_MOESM2_ESM.pdf]
